# Artificial Intelligence in Spine Neuroimaging: Diagnostic and Prognostic Utility of Novel Biomarkers in Lower Back Pain

**DOI:** 10.3390/jcm15124447

**Published:** 2026-06-09

**Authors:** Danai Stefanou, Ornella Moschovaki-Zeiger, Georgios Charalampopoulos, Nikolaos-Achilleas Arkoudis, Evgenia Efthymiou, Georgios Velonakis, Nikolaos Kelekis, Dimitrios K. Filippiadis

**Affiliations:** 2nd Department of Radiology, University General Hospital “ATTIKON”, Medical School, National and Kapodistrian University of Athens, 12462 Athens, Greece; danaistefanou@hotmail.com (D.S.);

**Keywords:** artificial intelligence, machine learning, lower back pain, lumbar spine, magnetic resonance imaging, imaging biomarkers, radiomics

## Abstract

Lower back pain (LBP) is a leading cause of disability globally, characterized by multifactorial origins that complicate accurate diagnosis and effective treatment planning. Artificial intelligence (AI), including machine learning (ML), deep learning (DL), and radiomics, has shown promise for improving the reproducibility and quantitative assessment of spine neuroimaging. This narrative review synthesizes current evidence on AI-derived imaging biomarkers in magnetic resonance imaging (MRI) and computed tomography (CT), with emphasis on disc degeneration, spinal stenosis, endplate signal abnormalities, paraspinal muscle composition, vertebral fractures, and spinal alignment. AI-based reconstruction, segmentation, and classification methods may reduce reader variability and enable standardized quantification of imaging features. However, the current evidence base remains dominated by technical and retrospective validation studies, and high diagnostic performance should not be interpreted as proof of improved patient-centered outcomes. The present review distinguishes technical feasibility, diagnostic assistance, prognostic association, and clinical utility, and highlights the persistent efficacy-effectiveness gap in AI-based spine imaging. Although multimodal models integrating imaging, clinical, biomechanical, and patient-reported data may improve future risk stratification, clinical translation remains constrained by heterogeneous datasets, limited external validation, incomplete interpretability, and evolving regulatory frameworks. Prospective multicenter validation and outcome-linked evaluation are required before AI-derived imaging biomarkers can be considered established tools for routine LBP management.

## 1. Introduction

Lower back pain (LBP) constitutes a substantial public health concern and remains one of the most common musculoskeletal conditions contributing to disability on a global scale [1,2]. Recent epidemiological evidence from the Global Burden of Disease 2021 analysis indicates that approximately 619 million individuals were affected globally in 2020, with projections suggesting an increase to 843 million affected individuals by 2050 [1].

Beyond its clinical impact, LBP imposes a substantial socioeconomic burden at both population and individual levels. Evidence consistently indicates that LBP disproportionately affects socioeconomically disadvantaged groups, contributing to reduced quality of life, impaired work capacity, and long-term disability [3]. The economic impact is considerable, with annual direct and indirect costs estimated at approximately US$2.2 billion in Brazil and exceeding US$130 billion in the United States, largely driven by productivity losses and work absenteeism [4,5]. Together, these findings emphasize the need for improved approaches to early identification, risk stratification, and individualized management of LBP.

Neuroimaging, particularly magnetic resonance imaging (MRI), plays a central role in the clinical evaluation of LBP. However, the diagnostic specificity of conventional imaging remains limited. Structural abnormalities such as disc degeneration, disc bulge or protrusion, facet joint degeneration, and Modic changes are frequently observed in symptomatic patients but are also highly prevalent among asymptomatic individuals, contributing to persistent discordance between imaging findings and clinical symptoms [6,7,8,9]. Moreover, qualitative image interpretation incompletely captures microstructural, inflammatory, and biochemical tissue alterations, such as annular fissuring, endplate microfractures, and inflammatory infiltration, that may be more closely linked to pain mechanisms and clinical trajectories [8,9].

Artificial intelligence (AI) has emerged as a promising approach to address these limitations by enabling objective, quantitative, and reproducible analysis of spine imaging data. Techniques such as radiomics, machine learning (ML), and deep learning (DL) facilitate the extraction of high-dimensional imaging features that capture tissue characteristics beyond visual interpretation [10,11]. By integrating imaging-derived biomarkers with clinical and biomechanical data, AI supports improved identification of pain-relevant pathology, prognostic stratification, and prediction of treatment response, while also demonstrating particular value in reducing inter-observer variability and standardizing measurements for interpretation tasks with high reader dependence, such as disc assessment, stenosis grading, endplate signal characterization, paraspinal muscle evaluation, and spinal alignment analysis. In this context, AI-based tools are best positioned as decision-support systems that augment radiologist interpretation, enable reproducible quantitative assessment at scale, and facilitate biomarker-guided, individualized care, rather than replacing clinical expertise [12,13,14,15,16,17,18].

The primary focus of this review is AI-derived imaging biomarkers in the context of lower back pain. Selected examples from adjacent spinal imaging domains, including trauma, deformity, and cervical degenerative disease, are discussed only where they illustrate relevant methodological principles, validation challenges, or translational considerations applicable to spine neuroimaging more broadly.

In the context of this review, imaging biomarkers are defined as quantifiable imaging-derived features that reflect underlying biological processes, structural alterations, or functional states, and that can be reproducibly measured and linked to clinically relevant outcomes.

The aim of this narrative review is to synthesize current evidence on the diagnostic, prognostic, and therapeutic utility of AI-derived imaging biomarkers in spine neuroimaging, with a specific focus on LBP. We highlight how AI-based methodologies address key limitations of conventional imaging, discuss their clinical applicability, and outline future directions necessary for their integration into routine spine care.

## 2. Methods

This review represents an expert-curated narrative synthesis rather than a systematic review and does not adhere to PRISMA reporting standards. Given the heterogeneity of study designs, imaging tasks, and reported outcomes, formal study-flow reporting and quantitative pooling were not performed. Selection bias is acknowledged as an inherent limitation. A targeted literature search was performed in PubMed/MEDLINE, Scopus, Web of Science, and Google Scholar for peer-reviewed English-language publications from January 2007 to April 2026. The search strategy combined terms related to the clinical condition, imaging modality, and AI method, including “lower back pain”, “low back pain”, “lumbar spine”, “spine MRI”, “computed tomography”, “spinal stenosis”, “disc degeneration”, “disc herniation”, “Modic changes”, “endplate”, “paraspinal muscle”, “vertebral fracture”, “spinal alignment”, “artificial intelligence”, “machine learning”, “deep learning”, “convolutional neural network”, “U-Net”, “transformer”, “radiomics”, “segmentation”, “classification”, “biomarker”, “prognosis”, “outcome prediction”, and “decision support”. Boolean combinations were adapted to each database, and reference lists of relevant reviews and original studies were screened to identify additional eligible articles. The search was updated in April 2026 to include recently published studies and datasets relevant to lumbar spine segmentation, reporting efficiency, and external validation resources [19,20,21].

Eligible publications included original research studies, validation studies, systematic or narrative reviews, regulatory documents, and dataset papers addressing AI-based acquisition, reconstruction, segmentation, classification, radiomics, prognostic modeling, workflow support, or translational implementation in spine imaging, with priority given to studies directly relevant to lower back pain and lumbar spine MRI/CT. Animal studies, editorials, case reports, commentaries, preprints, and thematically unrelated articles were excluded. Studies were qualitatively appraised according to validation design, dataset size, methodological transparency, clinical relevance, interpretability, endpoint type, and consistency between reported findings and cited evidence. Particular emphasis was placed on distinguishing technical performance, diagnostic assistance, prognostic association, and clinical utility, because these categories represent different levels of translational evidence. Supplementary Table S1 summarizes sample size/dataset, imaging modality, AI task, validation strategy, qualitative design-level quality/risk-of-bias appraisal, applicable reporting guidance, and translational evidence level.

## 3. Review of Current Literature

### 3.1. AI in Diagnostic Spinal Imaging

#### 3.1.1. Acquisition and Reconstruction as Determinants of Diagnostic Quality

AI is increasingly applied at early stages of the spine imaging pipeline, with clinical value emerging not only from downstream classification tasks but also from upstream processes such as image acquisition and reconstruction. DL–based reconstruction techniques improve image quality by reducing noise and motion-related artifacts and by enhancing acquisition efficiency, thereby providing a more consistent and standardized foundation for subsequent segmentation, quantification, and classification tasks in spine imaging [14,18].

Across lumbar spine MRI studies, DL–based reconstruction methods have consistently been associated with measurable improvements in image signal-to-noise characteristics, on the order of approximately 30%, while maintaining anatomical fidelity across varying field strengths and acquisition protocols, alongside gains in spatial resolution and perceived sharpness derived from learned volumetric feature relationships [22,23,24]. In lumbar spine computed tomography (CT), DL–enhanced reconstruction has demonstrated significantly lower image noise compared with standard reconstruction and has enabled radiation dose reductions of up to 72% while maintaining diagnostic image quality [25,26]. In a prospective, randomized, multicenter study, DL image enhancement maintained perceived diagnostic spine MRI quality despite a 40% scan-time reduction, with overall image quality rated statistically equivalent or subjectively superior to standard-of-care across features including signal-to-noise ratio (SNR), artifact burden, and multiple pathology categories [27]. Collectively, these findings support AI-driven acquisition and reconstruction as clinically meaningful enablers of diagnostic quality, efficiency, and standardization in routine spine imaging, consistent with practical implementation frameworks in spine neuroimaging [18,22,23,24,25,26].

Reported performance metrics such as accuracy, Dice coefficient, kappa agreement, or AUC should be interpreted in relation to task type, validation design, disease prevalence, calibration, and deployment context. These metrics should not be considered proxies for clinical benefit in the absence of prospective outcome-based validation [28].

Additional clinical evaluation confirms that deep learning image processing can reduce spine MRI scan time by 40% while maintaining diagnostic integrity and image quality, although these data remain focused on image quality and workflow efficiency rather than patient-level outcomes [27].

#### 3.1.2. Automated Segmentation to Reduce Workload and Inter-Reader Variability

A consistent diagnostic limitation in spine imaging is the time burden and inter-reader variability of manual segmentation. AI-based segmentation—most commonly using DL architectures—can delineate vertebrae, discs, spinal canal, and adjacent structures more efficiently and with improved reproducibility, supporting standardized reporting and quantitative measurement. These tools are particularly important where small differences in region-of-interest placement can alter downstream metrics (e.g., cross-sectional area measurements, fat fraction estimates, or radiomic features). A growing number of studies highlight this as a practical step toward reducing inter-rater variability and enabling scalable quantitative workflows under radiologist supervision [18]. In medical image segmentation, convolutional neural networks (CNNs) learn hierarchical image representations directly from pixel data, eliminating the need for handcrafted feature extraction and enabling end-to-end optimization for anatomical delineation tasks [29]. AI models used for automated spine segmentation include two-dimensional encoder–decoder architectures such as U-Net, which operate on slice-based inputs, three-dimensional convolutional neural networks (3D CNNs) that leverage volumetric context for improved spatial consistency, and transfer-learning approaches that adapt pre-trained networks to spine anatomy to improve performance and generalizability across heterogeneous datasets. To support generalizability across anatomy and scanner heterogeneity, multi-vendor CT segmentation datasets incorporating anatomic variation have been developed as shared resources for model training and validation [30]. U-Net–based architectures, which use a two-dimensional encoder–decoder design to perform slice-wise segmentation with precise spatial localization, are widely used in spine imaging. At the level of representative performance benchmarks, U-Net–based disc segmentation on spine MRI has achieved Dice scores around 0.85 [31]. 3D CNNs, which operate on volumetric image inputs and explicitly model inter-slice spatial context, have been reported to achieve Dice values > 0.90 across multiple complex spine structures in multi-tissue segmentation tasks [32,33], with higher computational and data requirements reported relative to slice-based approaches. Transfer learning in which models pretrained on large external datasets or related anatomic tasks are fine-tuned for spine segmentation, has been applied in spine imaging to address limited labeled data availability and support model generalization [34]. More generally, deep transfer learning applies weight parameters from a pre-training model and then trains on clinician-labeled MRI datasets to adapt the model to the target domain [34]. Beyond standard CNN segmentation pipelines, transformer–CNN hybrid architectures (e.g., Symmetric Transformer–CNN (SymTC) [35]) and diffusion-based segmentation models (e.g., SpineSegDiff) [13] have been described for multi-structure spine segmentation, with the goal of improving robustness to protocol heterogeneity and cross-dataset consistency in spine MRI, although hybrid models are associated with higher computational complexity and remain limited in routine clinical availability, and diffusion-based approaches remain emerging with limited large-scale clinical validation [18].

Reported agreement with expert readers should be interpreted in the context of study design, as many models are validated against single-reader annotations or limited consensus standards, which may themselves exhibit inter-observer variability.

Consistent with this cautious interpretation, a 2026 systematic review of deep learning for lumbar spine MRI segmentation found that U-Net and U-Net-derived architectures remain the most frequently used approaches, but also emphasized that many studies still rely on private datasets and that external validation remains uncommon [19].

#### 3.1.3. Classification and Diagnosis of Spine Pathologies

##### Disc Degeneration and Intervertebral Disc Pathology

Intervertebral disc degeneration is among the most common structural correlations of LBP and is conventionally assessed using visual grading systems such as Pfirrmann grading. While widely adopted, these systems remain susceptible to inter-observer variability and provide limited biological granularity, particularly for detecting subtle or longitudinal changes in disc tissue characteristics [36]. To address these limitations, AI-supported approaches have been applied to lumbar spine MRI to derive more objective and reproducible disc-related imaging descriptors. In this setting, radiomics-based analysis uses quantitative image-derived descriptors, including intensity- and texture-based measures capturing spatial appearance and heterogeneity, which are subsequently modeled using machine-learning algorithms such as Random Forest, Adaptive Boosting (AdaBoost), or Logistic Regression to support prediction or classification tasks [37,38,39].

Feature selection in radiomics typically involves dimensionality reduction techniques such as least absolute shrinkage and selection operator (LASSO), principal component analysis, or recursive feature elimination to mitigate overfitting and improve generalizability. Feature fusion may be implemented through early integration (feature concatenation), intermediate representation learning, or late fusion strategies combining independent model outputs.

In disc herniation and degenerative cohorts, radiomics-based approaches have been used to extract quantitative intensity-, shape-, and texture-derived features from segmented intervertebral discs on routine MRI and to analyze these features using conventional machine-learning models [10]. In a representative surgical cohort of patients undergoing lumbar discectomy, incorporation of three-dimensional radiomics features from T2-weighted MRI yielded minimal but detectable improvement in predictive performance compared with clinical variables alone, with mean test accuracies of approximately 88% across evaluated models. These findings suggest that radiomics features capture aspects of disc phenotype not fully reflected by qualitative visual assessment, although their incremental clinical value appears context dependent. By contrast, DL methods in medical imaging commonly use convolutional neural networks (CNNs), which are widely applied computer-vision architectures that learn task-relevant image representations directly from pixel data for classification/segmentation pipelines [39].

Complementarily, DL classifiers, most commonly convolutional neural networks implemented using standard architectures such as Residual Network (ResNet) or GoogleNet-derived backbones, have been developed for automated intervertebral disc degeneration grading directly from MRI. In a large multicenter study comprising 1599 patients and 7948 lumbar discs, a deep convolutional neural network trained for automated Pfirrmann grading achieved near-expert reliability (Cohen’s kappa coefficient κ = 0.92), an average sensitivity of 90.2%, precision of 92.5%, and a mean absolute error of 0.08 Pfirrmann grades, with 99.2% of predictions deviating by no more than one grade from expert annotation. These results compare favorably with previously reported human inter- and intra-observer agreement for conventional Pfirrmann grading systems, although this should not be interpreted as a direct head-to-head comparison with expert consensus on the same dataset [40,41].

AI has also been applied to disc herniation identification using cascade convolutional neural network architectures, in which the diagnostic task is decomposed into sequential stages. In this framework, an initial DL model automatically detects and localizes individual lumbar intervertebral discs and extracts disc-specific regions of interest from axial and sagittal MRI. These localized disc regions are then passed to subsequent convolutional neural networks dedicated to disc-level herniation classification, with optional decision fusion across image planes to improve robustness. Using this cascade strategy, automated systems have demonstrated high disc-level detection and classification performance, with reported disc-level classification accuracies exceeding 97% in retrospective evaluations and improved consistency of disc-level labeling compared with single-stage approaches [42]. By separating disc localization from pathology classification, cascade CNN architectures reduce error propagation and support more standardized identification of disc herniation in routine lumbar MRI interpretation [42].

##### Spinal Canal Stenosis and Quantitative Dural Sac Assessment

Lumbar spinal stenosis assessment is often limited by subjective grading and inter-reader variability. DL systems have been used to automate central canal and foraminal stenosis grading and to support objective canal quantification. In particular, fully automated segmentation approaches for dural sac cross-sectional area (DSCA)—a clinically interpretable quantitative biomarker—enable standardized and reproducible quantification of canal dimensions on lumbar MRI across examinations and institutions [12,43]. Including MultiResUNet-based implementations, automated DSCA methods have demonstrated very high agreement with expert measurements (reported Pearson correlation ~0.99) and low absolute error, supporting standardized assessment and longitudinal tracking in routine lumbar MRI [12,43]. Several DL–based frameworks, including DeepSpine [44] and SpineNet [45], have been developed to automate lumbar spinal stenosis grading on MRI by learning severity labels from expert-annotated datasets, enabling large-scale, reproducible assessment of canal narrowing. Beyond DSCA quantification, DL-based stenosis grading systems have demonstrated moderate performance for ordinal four-class grading, with reported average accuracies of 70.6% for central canal stenosis and 67.1% for neural foraminal stenosis in the DeepSpine model [44]. In the SpineNet framework, ordinal agreement with an expert radiologist was 65.7%, while dichotomous grading achieved 94% agreement (κ = 0.75) compared with an experienced orthopedic surgeon [45,46].

##### Modic Changes and Vertebral Endplate Abnormalities

Modic changes (MCs) are vertebral endplate-adjacent marrow signal abnormalities associated with LBP, particularly type 1 lesions [7,47]. Conventional MC grading is semiquantitative and can be vulnerable to variability, especially across heterogeneous imaging protocols [48]. Quantitative and AI-assisted strategies have therefore focused on (i) improving reliability via feature-based measures and (ii) automated detection/classification. Early quantitative approaches using morphology and signal features improved intra-/inter-rater agreement versus unassisted reads [49]. More recent DL approaches have automated MC detection/mapping with strong concordance to expert annotation, including voxel-wise strategies designed to capture mixed lesions and preserve tissue context, clinically relevant given the frequency of heterogeneous MC phenotypes [50,51], although voxel-wise implementations can be complex and remain of limited routine clinical availability [50,51,52]. A representative framework combining lesion localization and classification (Single-shot multibox detector (SSD) + 18-layer Residual Network (ResNet18)) reported accuracy ~86% and κ ~0.7 agreement approximating expert performance [53]. Because mixed Modic phenotypes are frequently observed (27.2% classified as mixed), voxel-wise DL mapping is particularly well suited to capturing transitional and heterogeneous lesions that are poorly represented by categorical grading schemes [52]. Voxel-wise detection analyses showed that predicted Modic changes were predominantly distributed at L4–S1 (74.4%), closely matching radiologist annotations (78.8%) and prior prevalence estimates [52]. AI-assisted Modic predictions have also been shown to improve agreement between junior and senior subspecialty radiologists, supporting their role in reducing reader-dependent variability in endplate assessment [18]. Automated models most commonly prioritize Modic types 1 and 2 due to their prevalence and the reported association of type 1 changes with nonspecific low back pain [7,18].

##### Lateral Recess Stenosis and Facet Arthropathy

Evaluation of lateral recess stenosis and facet arthropathy on lumbar MRI is clinically relevant but often challenged by reader-dependent variability and inconsistent reporting. In a large retrospective study, Hallinan and colleagues demonstrated that a DL model achieved almost-perfect agreement with subspecialist radiologists for dichotomous classification of lateral recess stenosis (normal/mild vs. moderate/severe), with κ values of 0.92, 0.95, and 0.92 for the musculoskeletal reference reader and two subspecialist radiologists, respectively [54]. Agreement for neural foraminal stenosis was slightly lower but remained high, with κ values of 0.94, 0.95, and 0.89, reflecting the greater anatomic complexity of foraminal assessment [54]. Building on these findings, these DL stenosis-grading frameworks have also been extended to adjacent degenerative features such as facet arthropathy, and interpretable two-stage framework incorporating automated localization and classification reported localization Dice scores well above 0.90 for key structures and area under the receiver operating characteristic (AUROC) curve values of 0.92 for neural foraminal stenosis and 0.93 for facet arthropathy, extending automated assessment to a pathology reported in 14–45% of chronic low back pain cases and frequently under-described in routine lumbar MRI reports [18].

##### Spine Trauma and Vertebral Fractures

Acute traumatic injuries of the spine constitute a severe imaging domain in which diagnostic delays can directly influence neurologic outcomes and management decisions. AI has been explored to enhance fracture detection, standardize volumetric image interpretation, and support radiologist decision-making in time-sensitive CT and radiographic workflows [55,56,57,58,59].

##### Osteoporotic Vertebral Fractures

Early applications of AI in this domain have focused on automated analysis of CT examinations for osteoporotic vertebral fracture detection. Tomita and colleagues developed a deep neural network trained on routine CT scans and evaluated it in a retrospective cohort of 1432 examinations, reporting an accuracy of 89.2% and an F1 score of 90.8% for fracture detection. While overall performance was comparable to that of practicing radiologists, human readers demonstrated higher specificity, supporting the role of AI as a pre-screening and case-flagging tool rather than an autonomous diagnostic system [55]. Complementary work by Burns and colleagues extended AI applications beyond detection to fracture classification and bone density assessment. Their automated framework achieved a sensitivity of 95.7% for fracture detection and localization, with a false-positive rate of 0.29 per patient and demonstrated high agreement with radiologist annotations for Genant fracture type classification (weighted κ = 0.90). In parallel, the system quantified vertebral body attenuation, identifying significantly lower values in patients with fractures compared with controls, thereby enabling opportunistic assessment of skeletal fragility from standard CT imaging [56].

##### Traumatic Thoracolumbar Fractures

AI-based approaches have also been applied to traumatic thoracic and lumbar fractures using both radiographs and CT. Murata and colleagues evaluated a convolutional neural network trained on anteroposterior and lateral thoracolumbar radiographs from 300 patients and reported an accuracy of 86% and a sensitivity of 84.7% for vertebral fracture detection. Model performance was non-inferior to that of orthopedic surgeons and demonstrated higher sensitivity than that of orthopedic residents, supporting potential utility in initial triage and screening settings [57]. CT-based systems allow more detailed vertebra-level analysis. For detecting, localizing, and classifying traumatic thoracic and lumbar vertebral fractures on CT, Burns and colleagues developed an automated method, achieving a sensitivity of 0.81 with a false-positive rate of 2.7 per examination. Analysis of model errors indicated that false positives were most frequently related to nutrient foramina, whereas false negatives commonly involved subtle fracture lines oriented parallel to vertebral endplates or adjacent to degenerative changes, highlighting persistent challenges in automated interpretation of complex trauma imaging [58].

##### Traumatic Cervical Spine Injuries

Given the high morbidity associated with missed cervical spine injuries and the central role of CT in acute trauma evaluation, AI algorithms have also been assessed in this setting. Small and colleagues evaluated a convolutional neural network for cervical spine fracture detection on 665 CT examinations, reporting a sensitivity of 76% and an overall accuracy of 92%. In comparison, fellowship-trained neuroradiologists achieved sensitivities of 93% and accuracies of 96%. Although AI performance was lower than expert readers, the system provided substantially faster time-to-alert and demonstrated miss patterns that closely overlapped with those of radiologists. Notably, several fractures detected by the AI system were initially overlooked during human interpretation, supporting the role of AI as a complementary safety and workflow prioritization tool rather than a standalone diagnostic solution [59].

##### Paraspinal Muscle Morphology and Composition

Paraspinal muscle morphology, particularly fatty infiltration of the multifidus and erector spinae, is a common imaging finding in patients with LBP and is most pronounced at lower lumbar levels [60,61]. Associations have been reported between paraspinal fatty degeneration, altered biomechanics, and chronic lumbar pathology, although conventional assessment remains limited by the time burden and inter-reader variability of manual segmentation, which can undermine consistency of muscle characterization [60,62].

MRI-based cohort studies indicate that increased paraspinal muscle fatty infiltration may be present even in the absence of significant differences in total muscle cross-sectional area, emphasizing muscle composition rather than size alone as a key imaging descriptor [9]. In one controlled study of patients with recurrent unilateral LBP in clinical remission, lean muscle fatty infiltration remained significantly elevated at lower lumbar levels and correlated with episode frequency (coefficient of determination, R^2^ ≈ 0.45), highlighting the ability of composition-sensitive measures to capture reproducible structural differences not readily appreciated on qualitative visual assessment [9].

AI-based approaches, most commonly DL segmentation models such as U-Net-derived architectures, enable automated delineation of paraspinal muscles and objective quantification of cross-sectional area and fat fraction with high reproducibility [15]. By reducing operator dependence and enabling standardized measurement, these tools support consistent and anatomically interpretable characterization of paraspinal muscle morphology and composition in routine lumbar MRI, aligned with established radiological workflows [63].

##### Spinal Alignment and Postural Parameters

Sagittal and coronal spinal alignment parameters, including lumbar lordosis, pelvic tilt, and sagittal vertical axis, are established radiological descriptors in degenerative spine disease and deformity assessment. These parameters are routinely used to characterize spinal posture and global balance on radiographs and MRI. However, manual measurement is time-consuming and subject to substantial interobserver variability, limiting reproducibility across readers and longitudinal assessments.

AI-based alignment assessment tools have demonstrated high agreement with expert measurements and improved measurement consistency, enabling standardized and reproducible quantification of alignment parameters across imaging modalities and populations [64,65,66]. The availability of U.S. Food and Drug Administration (FDA)-approved implementations in deformity-related contexts further supports the technical maturity and translational readiness of AI-enabled alignment analysis for clinical imaging applications [17]. End-to-end DL systems for automated alignment analysis have achieved low absolute error in key parameters, including reported Cobb angle mean absolute error of approximately 4° across anteroposterior and lateral views, and intraclass correlation coefficient (ICC) exceeding 0.95 compared with expert measurements [67,68]. These performance metrics indicate that AI-enabled tools can reliably reproduce standard alignment measurements with reduced reader-dependent variability.

By automating landmark detection and parameter calculation, AI-based alignment assessment strengthens the diagnostic characterization of spinal posture by improving measurement reliability and standardization in routine and research spine imaging [67,68]. Beyond conventional radiographic alignment, additional machine learning applications include scoliosis classification and curve characterization using surface topography and automated landmark detection, reflecting a broader shift toward objective and reproducible measurement pipelines in spine assessment [66,67,68,69,70,71].

A structured overview of representative AI models and approaches discussed in spine neuroimaging, together with their current evidence role, principal advantages, and limitations, is provided in Table 1.

Table 1 provides a methodological overview of representative AI approaches; however, evidentiary strength and translational maturity vary substantially across applications and are further summarized in Supplementary Table S1.

### 3.2. Prognostic Association and Potential Decision-Support Value of AI-Derived Imaging Biomarkers

One of the longstanding challenges in LBP management is the inconsistent relationship between structural imaging findings and clinical outcomes such as pain trajectory, disability, or response to treatment. Mechanistic radiology literature underscores that pain generation reflects heterogeneous biological pathways, including mechanical compression, inflammation, biochemical mediators, and altered load distribution, which helps explain why conventional “structure-only” interpretations may not reliably predict outcome [8]. Against this background, AI-derived imaging biomarkers aim to quantify imaging phenotypes more reproducibly and associate them with longitudinal outcomes (e.g., pain, function, progression, and recovery), thereby enabling risk-informed stratification beyond purely descriptive categorization [74].

Accordingly, AI-derived imaging biomarkers should currently be regarded as tools with potential diagnostic or prognostic assistance rather than validated instruments for treatment optimization, unless supported by prospective outcome-based evidence.

Technical improvements in image acquisition, segmentation, or classification may improve reproducibility and efficiency, but they do not necessarily establish patient-level benefit unless linked to validated clinical outcomes.

Diagnostic performance, prognostic association, and therapeutic decision-support utility represent distinct levels of evidence and should not be conflated. Current AI applications in spine imaging are strongest for technical and diagnostic assistance, whereas evidence for outcome-guided therapeutic decision-making remains limited.

Recent prospective validation of an AI model for lumbar surgery outcomes has reported a receiver operating characteristic score of 0.816 and classification accuracy of 70% for prediction of minimal clinically important difference, illustrating progress toward outcome-linked validation while also underscoring that prospective interventional trials comparing AI-guided care with standard care remain absent [75].

While AI-derived imaging biomarkers may support prognostic modeling, most available evidence remains based on retrospective associations or technical validation studies. These findings should therefore be interpreted as hypothesis-generating or decision-support oriented rather than as proof of improved patient outcomes.

A critical limitation across the current literature is the efficacy-effectiveness gap, whereby strong performance in retrospective datasets does not necessarily translate into improved patient outcomes. Prospective interventional studies evaluating clinical impact remain limited.

Improved structural characterization alone may not resolve the discordance between imaging findings and clinical symptoms in LBP, as pain generation reflects multifactorial biological, biomechanical, and psychosocial processes. This underscores the need for multimodal models that integrate imaging-derived features with clinical, functional, and patient-reported data.

As a methodological comparator rather than an AI-derived biomarker, functional imaging evidence illustrates the type of outcome-linked validation that imaging biomarkers require before they can inform treatment selection. Vega-Alvear et al. evaluated SPECT/SPECT-CT for facet-intervention response in mechanical lower back pain; across six studies (*n* = 308), positive SPECT findings were associated with greater post-intervention pain relief (RR 2.06, 95% CI 1.54–2.75), whereas SPECT-CT evidence was less consistent [74]. This example supports the principle of outcome-linked imaging stratification, but it should not be interpreted as validation of AI-derived biomarkers or AI-guided management.

For MRI-derived paraspinal muscle and degenerative biomarkers, current evidence mainly supports quantitative measurement and biological plausibility. The available AI literature remains strongest for reproducible segmentation, classification, and feature extraction, while evidence that AI-derived biomarkers improve treatment selection or patient outcomes remains limited [15,17,18,76].

From a clinical translation perspective, AI-derived imaging biomarkers should be considered candidate inputs to prognostic models rather than stand-alone tools for treatment selection. Quantitative imaging stratification may help generate hypotheses about pain mechanisms when interpreted with clinical context, but evidence that such stratification improves treatment matching remains limited [8]. Studies integrating imaging and clinical features suggest that AI models can predict selected functional endpoints such as disability scores, complications, or return-to-work status, with reported predictive accuracies in the range of approximately 70–85% in selected cohorts [11,17,77]. These findings support the feasibility of prognostic modeling, but do not yet establish therapeutic benefit or outcome-improving clinical deployment.

Beyond imaging alone, future multimodal decision-support frameworks may integrate imaging-derived biomarkers with electronic health records, patient-reported outcome measures (PROMs), biomechanical data, and non-imaging risk signals—including genetic, lifestyle, and occupational factors—to support risk stratification and outcome prediction. At present, however, these approaches should be regarded as investigational unless prospectively validated in clinically defined LBP pathways [72,73,78].

Genetically informed evidence supports the relevance of non-imaging risk signals for future multimodal models: Mendelian randomization syntheses report positive associations between back pain risk and lifestyle-related exposures including smoking (Odds ratio (OR) ~1.30), higher BMI (OR ~1.18), alcohol consumption (OR ~1.31), and sleep disturbance/insomnia (OR ~1.38) [79]. Occupational meta-analytic evidence from professional driver cohorts similarly indicates elevated LBP risk in association with prolonged working time (>10 h/day; OR ~2.49), longer driving duration (>5 years; OR ~2.12), lack of back support (OR ~1.81), high work-related pressure (OR ~2.04), and job dissatisfaction (OR ~1.57), reinforcing the potential value of exposure context alongside imaging phenotypes [80]. Together, these findings provide rationale for evaluating multimodal AI approaches that combine imaging-derived biomarkers with clinical, biomechanical, genetic, and exposure-linked variables for prognostic stratification; they do not by themselves demonstrate improved treatment selection [72,79,80].

Potential future applications of validated imaging biomarkers span several LBP management settings, including conservative care, minimally invasive/interventional procedures, and operative pathways. Current evidence is strongest for reproducible measurement and selected prognostic associations; therefore, use for patient selection, treatment monitoring, or pathway selection should be considered investigational until confirmed by prospective outcome-linked studies [17,74,75,76,81]. Across these settings, near-term value is most likely when AI-derived biomarkers are transparent, reproducible, and interpreted under clinician supervision.

### 3.3. Clinical Applicability and Integrated Clinical Value of AI in Spine Neuroimaging

A key clinical advantage of AI in spine neuroimaging is its ability to support standardized image interpretation for tasks known to exhibit substantial inter-observer variability, including stenosis grading, endplate signal characterization, paraspinal muscle assessment, and alignment quantification [77,82,83]. AI-based tools enable reproducible, quantitative assessment of these features, but their impact on diagnostic or treatment decisions remains dependent on validation setting, intended use, and clinical oversight.

For clinical translation, evidence should be interpreted according to intended use and validation level, with clear separation between technical feasibility, retrospective diagnostic validation, prognostic association, and demonstrated impact on patient-centered outcomes.

The clinical relevance of AI models should be interpreted according to intended use, distinguishing workflow optimization, quantitative measurement, diagnostic assistance, prognostic modeling, and patient-level decision support.

Most AI systems discussed remain in the research or early validation phase and should be implemented, if at all, as radiologist-supervised decision-support tools rather than autonomous diagnostic systems.

From a regulatory perspective, currently FDA-cleared AI systems in spine imaging are largely limited to narrow assistive applications, including triage/notification tools and quantitative measurement systems, rather than autonomous diagnostic or therapeutic platforms. For example, CINA-CSpine (Avicenna.AI) is FDA-cleared under the 510(k) pathway for triage and notification of suspected cervical spine fractures on CT, enabling prioritization of urgent findings for radiologist review rather than providing a definitive diagnosis. Similarly, AI-based tools for spinal alignment and parameter measurement are available to support reproducible quantification of radiographic features, but do not establish diagnostic conclusions or guide treatment decisions. At present, there are no FDA-approved AI systems for diagnosing lower back pain, identifying specific pain generators, predicting treatment response, or directing clinical management. Accordingly, the current role of AI in spine imaging should be understood as augmentative and workflow-oriented, and its impact on patient outcomes remains to be established [84].

From an implementation perspective, the most robust clinical models emphasize radiologist-in-the-loop deployment, in which deep learning systems generate structured, semi-automated assessments that are reviewed and, when necessary, modified prior to report finalization [18,82,83]. Deep learning-assisted reporting has been shown to reduce interpretation time while maintaining or improving inter-observer agreement, with particularly pronounced benefits among general and in-training radiologists, thereby enhancing reporting efficiency, objectivity, and consistency without displacing clinical judgment or accountability. AI-assisted standardization may be especially valuable in settings with limited access to subspecialty expertise, where structured first-pass assessments can highlight regions of interest while preserving clinician oversight and clear accountability structures [72,77,82].

A recent 2026 preliminary study further supports the workflow-oriented role of AI in lumbar spine MRI reporting, showing that AI-assisted interpretation shortened reporting time; however, this evidence relates primarily to efficiency and should not be interpreted as proof of improved patient-level outcomes [20].

**Figure 1:**
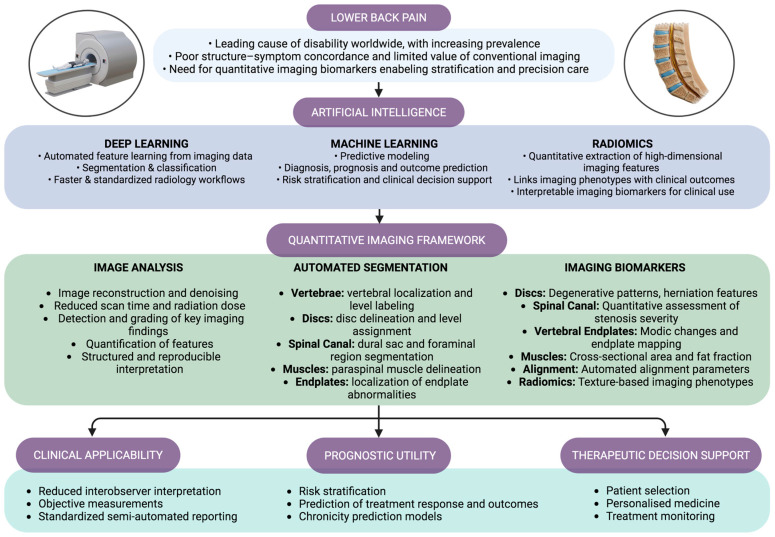
Conceptual overview of AI workflow in lower back pain imaging. The figure illustrates potential stages of AI-supported image acquisition, quantitative biomarker extraction, validation, and radiologist-in-the-loop interpretation. Potential downstream decision-support applications are shown as future, validation-dependent pathways and do not imply established routine clinical implementation.

Across diagnostic applications, segmentation-driven deep learning architectures are often closest to clinical translation because they generate anatomically interpretable outputs aligned with established radiological concepts and reporting practices while enabling reproducible quantitative measurement at scale [18]. An overview of the AI workflow in spine neuroimaging, encompassing image acquisition, quantitative biomarker extraction, validation, and radiologist-in-the-loop interpretation, is summarized schematically (Figure 1). By contrast, radiomics-based machine learning approaches offer distinct but complementary value, particularly in prognostic modeling by enabling extraction of high-dimensional features associated with pain severity, disability, and longitudinal outcomes. However, these models remain more sensitive to acquisition variability and typically offer lower interpretability than segmentation-based outputs, limiting their robustness across scanners and clinical settings. Accordingly, the relative clinical utility of segmentation- versus radiomics-based models is task-dependent rather than hierarchical, with optimal model selection driven by the intended diagnostic, prognostic, or therapeutic application, consistent with the model-specific advantages and limitations summarized in Table 1.

Segmentation-based approaches are generally preferred for anatomically interpretable tasks and quantitative measurement, whereas radiomics-based models may provide additional prognostic information through high-dimensional feature extraction, albeit with greater sensitivity to acquisition variability.

## 4. Limitations and Future Directions

Despite rapid methodological progress, translation of AI-derived imaging biomarkers into routine spine care remains constrained by several interrelated challenges. A central limitation is the lack of robust multicenter validation across heterogeneous imaging environments. Variability in scanner hardware, acquisition protocols, and reconstruction pipelines can substantially alter both radiomic feature distributions and deep learning model performance. Accordingly, meaningful validation requires prospective evaluation across diverse institutions using standardized annotation protocols and clinically relevant endpoints. Harmonization strategies, including statistical approaches such as ComBat, have been proposed to mitigate scanner-related variability in radiomic features across scanners and acquisition protocols; however, these methods rely on assumptions regarding feature distribution and may attenuate biologically relevant signal if applied indiscriminately. Beyond feature-level harmonization, approaches such as domain adaptation, model recalibration, and federated learning may be required to support generalizability across institutions.

Explainability remains a critical barrier to clinical adoption. While saliency-based techniques (e.g., gradient-weighted class activation mapping) provide intuitive visualization of model attention, these methods may lack stability and are sensitive to input perturbations. Complementary approaches, including feature attribution methods (e.g., SHAP or LIME) and attention-based architectures, may offer more structured interpretability, particularly when aligned with anatomically meaningful regions such as intervertebral discs, vertebral endplates, and paraspinal musculature. Clinically useful explainability frameworks must therefore move beyond generic heatmaps toward anatomically grounded and reproducible representations.

From a regulatory perspective, most currently available AI systems in medical imaging are cleared under the FDA 510(k) pathway as software supporting triage, detection, or measurement tasks, rather than autonomous diagnostic or therapeutic decision-making. Advancement toward prognostic modeling or treatment guidance will require prospective, outcome-based validation and may necessitate adaptive regulatory frameworks for continuously evolving algorithms. Importantly, regulatory clearance reflects safety and performance within a defined intended use and does not establish clinical benefit.

Future research should prioritize multicenter datasets with harmonized acquisition and annotation standards, transparent validation and calibration reporting, and integration of imaging-derived biomarkers with clinical, biomechanical, and patient-reported data. Reporting should follow design-matched EQUATOR guidance, including CLAIM and STARD-AI for AI imaging and diagnostic-accuracy studies, TRIPOD+AI/TRIPOD-AI for prediction models, STROBE for observational cohorts, PRISMA/PRISMA-ScR for evidence syntheses, and CONSORT-AI/SPIRIT-AI for interventional trials and protocols. Prospective studies are needed to determine whether AI-assisted workflows improve diagnostic accuracy, efficiency, or patient-centered outcomes in lower back pain [17,18,72,78,84,85,86]. Publicly available curated datasets, including the 2026 LSS MRI AISSLab dataset for segmentation and foraminal stenosis annotation, may help improve transparency, benchmarking, and external validation, but they do not substitute for prospective clinical-outcome studies [21].

### Knowledge Gaps

Several knowledge gaps remain before AI-derived imaging biomarkers can be integrated into routine lower back pain pathways. First, most models have been trained and tested on retrospective or single-center datasets, with limited evidence that performance is preserved across scanners, institutions, patient populations, and acquisition protocols. Second, published studies frequently report technical metrics such as Dice score, AUC, accuracy, or kappa agreement, but comparatively few link AI-derived measurements to validated clinical endpoints such as pain persistence, disability, treatment response, surgical decision-making, return to work, or quality of life.

Third, the relative value of different biomarker classes remains insufficiently defined. Segmentation-based measurements are anatomically interpretable and closest to current reporting workflows, whereas radiomics and multimodal models may capture subtler tissue phenotypes but are more sensitive to feature instability, preprocessing choices, and domain shift. Fourth, there is limited evidence on how radiologists, spine surgeons, pain physicians, physiotherapists, and patients should interact with AI outputs in real-world care pathways. Finally, regulatory clearance, ethical governance, bias monitoring, and accountability frameworks must evolve alongside prospective validation so that AI systems are evaluated not only for diagnostic performance, but also for safety, equity, interpretability, workflow compatibility, and patient-centered benefit.

## 5. Conclusions

This narrative review summarizes current evidence on AI-derived imaging biomarkers in spine neuroimaging for lower back pain. The reviewed literature supports the ability of AI-based methods to improve image reconstruction, automate segmentation, standardize measurements, and assist classification of imaging findings such as disc degeneration, spinal stenosis, Modic changes, paraspinal muscle composition, vertebral fractures, and spinal alignment. These applications are most strongly supported as tools for technical measurement, workflow efficiency, and radiologist-supervised diagnostic assistance.

The evidence reviewed also indicates that AI-derived imaging biomarkers have potential prognostic value when integrated with clinical, biomechanical, and patient-reported data. However, this conclusion should be interpreted cautiously because most available studies remain retrospective, technically focused, or based on selected cohorts. High diagnostic accuracy, segmentation performance, or reporting efficiency does not by itself demonstrate improved patient outcomes, treatment selection, or personalized management.

Accordingly, AI in spine neuroimaging should currently be viewed as an augmentative decision-support framework rather than an established autonomous diagnostic or therapeutic tool for lower back pain. Prospective multicenter validation, transparent reporting, external dataset testing, radiologist-in-the-loop implementation, and outcome-linked evaluation are required before AI-derived imaging biomarkers can be considered established instruments for routine clinical decision-making in lower back pain care.

## Figures and Tables

**Table 1:** Representative AI approaches in spine neuroimaging: imaging application, current evidence role, advantages, and limitations.

AI Model/ Approach	Imaging Application	Evidence-Supported Role	Advantages	Limitations
DL-based image reconstruction and AI image enhancement [18,22,23,24,25,26,27]	Lumbar spine MRI and CT image reconstruction	Technical/measurement support	Improved image quality (e.g., SNR increases up to ~30%); reduced noise and motion artifacts; substantial scan-time reduction (40%) or radiation dose reduction (up to ~72%) while maintaining diagnostic quality; improved standardization across scanners	Vendor- and sequence-specific implementations; limited algorithm transparency; potential domain shift across scanners and protocols; requires validation against standard-of-care reconstruction
U-Net and U-Net-derived architectures [18,31,32,33]	Automated segmentation of vertebrae, intervertebral discs, spinal canal, paraspinal muscles (MRI, CT)	Technical/measurement support; diagnostic assistance	High segmentation performance for multiple spine structures (e.g., disc segmentation Dice around 0.85 reported for U-Net–based approaches; 3D CNN approaches have been reported to reach Dice values > 0.90 across multiple complex spine structures); substantial reduction of inter-observer variability; anatomically interpretable outputs aligned with radiological workflows; often implemented with transfer learning to improve generalizability	Performance dependent on training data quality; limited generalizability across scanners without retraining; limited explainability
MultiResUNet [12,43]	Dural sac segmentation and DSCA quantification (lumbar MRI)	Technical/measurement support; prognostic association; therapeutic decision support not established	Near-expert agreement for DSCA measurement (reported Pearson correlation ~0.99; low absolute error); objective and clinically interpretable stenosis biomarker; suitable for longitudinal monitoring	Mostly validated in retrospective cohorts; limited prospective and outcome-driven validation
Transformer–CNN hybrid models (e.g., SymTC) [18]	Vertebral and disc segmentation across heterogeneous MRI protocols	Technical/measurement support	Improved robustness to protocol variability; better generalization than CNN-only models	Higher computational complexity; limited availability in routine clinical practice
Diffusion-based segmentation models (e.g., SpineSegDiff) [18]	Multi-structure spine segmentation (MRI)	Technical/measurement support	Strong cross-dataset consistency; reduced sensitivity to acquisition heterogeneity	Emerging methodology; limited large-scale clinical validation
Radiomics + ML models (Random Forest, AdaBoost, Logistic Regression) [10,37,38,39]	Disc herniation/LBP-associated patterns (lumbar MRI)	Diagnostic assistance; prognostic association	Feature-level interpretability; effective discrimination of LBP-associated imaging patterns; suitable for moderate dataset sizes	Feature instability across scanners; need for standardized preprocessing; risk of overfitting
Deep CNN classifiers (e.g., ResNet, GoogleNet) [40,41]	Intervertebral disc degeneration characterization/LBP-associated imaging patterns (lumbar MRI)	Diagnostic assistance	Automated feature learning; improved discrimination/consistency vs. conventional qualitative assessment	“Black-box” behavior; limited explainability; requirement for large annotated datasets
Cascade CNN architectures for disc localization and herniation detection [42]	Automated disc-level labeling and disc herniation detection (lumbar MRI)	Diagnostic assistance	Supports more consistent disc-level labeling; may help link morphological disc abnormalities to nerve root compromise; improves workflow efficiency	“Black-box” behavior; lim-ited explainability; requirement for large annotated datasets
Deep learning–based Modic change detection and classification frameworks (SSD + ResNet18) [53]	Automated Modic change localization and type classification (lumbar MRI)	Diagnostic assistance	Good agreement with expert annotation (reported accuracy ~86%, κ ~0.7); automated lesion localization and classification approximating expert performance	Performance dependent on annotation quality; limited explainability; requires expert verification
Voxel-wise DL models for Modic changes [50,51]	Automated detection and mapping of Modic type heterogeneity (MRI)	Diagnostic assistance; prognostic association	Captures mixed Modic phenotypes; improved grading reproducibility; better tissue-level representation	Complex implementation; limited clinical availability
DL-based spinal stenosis grading classifiers (DeepSpine, SpineNet) [18,44,45,46,54]	Central canal, lateral recess, and neural foraminal stenosis grading; facet arthropathy assessment (lumbar MRI)	Diagnostic assistance	Automated and reproducible stenosis grading; moderate accuracy for four-class grading (≈65–71%; reported average accuracies 70.6% (central canal) and 67.1% (neural foraminal) in DeepSpine); high agreement for dichotomous classification (κ = 0.75); reduced inter-reader variability	Reduced performance for fine-grained severity differentiation; reliance on report-derived labels in some studies; limited prospective validation
DL-based paraspinal muscle analysis [15,63]	Muscle segmentation and fat fraction quantification (MRI)	Technical/measurement support; prognostic association not established	Objective, reproducible assessment of muscle composition; quantitative biomarkers that may be examined in relation to pain persistence and rehabilitation outcomes.	Risk of overinterpretation; causality not fully established; requires radiologist verification/clinical context
AI-based spinal alignment assessment tools [17,64,65,66,67,68]	Automated sagittal and coronal parameter measurement (radiographs, MRI)	Technical/measurement support; prognostic association	High agreement with expert measurements (ICC > 0.95); reduced measurement variability; FDA-approved implementations available	Predominantly validated in deformity populations; limited data in nonspecific LBP
DL fracture detection and classification models [55,56,57,58,59]	Vertebral fracture detection/localization and classification (CT, radiographs)	Diagnostic assistance; safety/workflow support	High sensitivity/specificity; reduced missed fractures; standardized trauma assessment (e.g., accuracy 89.2% and F1 90.8% for osteoporotic fracture detection; sensitivity 95.7% with false-positive rate 0.29/patient; cervical fracture detection sensitivity 76%, overall accuracy 92%)	Limited prospective validation; performance influenced by image quality and artifacts
Multimodal AI decision-support systems (CAD platforms) [18,72,73]	Integration of imaging-derived biomarkers with clinical data and PROMs	Prognostic modeling potential; therapeutic decision support not established	Feasible outcome prediction in selected cohorts; may inform future risk stratification after prospective validation.	Regulatory complexity; workflow integration challenges; governance and accountability issues; therapeutic benefit not yet established.

Abbreviations List: 3D: Three-dimensional; AdaBoost: Adaptive Boosting; AI: Artificial intelligence; CAD: Computer-aided diagnosis; CNN: Convolutional neural network; CT: Computed tomography; DL: Deep learning; DSCA: Dural sac cross-sectional area; F1: F1 score; FDA: U.S. Food and Drug Administration; ICC: Intraclass correlation coefficient; κ: Cohen’s kappa coefficient; LBP: Lower back pain; ML: Machine learning; MRI: Magnetic resonance imaging; PROMs: Patient-reported outcome measures; ResNet: Residual Network; ResNet18: 18-layer Residual Network; SNR: Signal-to-noise ratio; SSD: Single-shot multibox detector; SymTC: Symmetric Transformer–CNN (hybrid model); U-Net: U-shaped convolutional neural network architecture.

## Data Availability

All data analyzed in the present study are included in this article.

## Supplementary Materials

The following supporting information can be downloaded at: https://www.mdpi.com/article/10.3390/jcm15124447/s1, Table S1: Structured quality/risk-of-bias appraisal, translational evidence level, and interpretive role of representative AI applications in spine imaging.

## Abbreviations

The following abbreviations are used in this manuscript:

3D	Three-dimensional
AdaBoost	Adaptive boosting
AI	Artificial intelligence
AUC	Area under the curve
AUROC	Area under the receiver operating characteristic curve
BMI	Body mass index
CAD	Computer-aided diagnosis
CI	Confidence interval
CNN	Convolutional neural network
CT	Computed tomography
DALYs	Disability-adjusted life years
DCM	Degenerative cervical myelopathy
DL	Deep learning
DSCA	Dural sac cross-sectional area
F1	F1 score
FDA	U.S. Food and Drug Administration
GBD	Global Burden of Disease
ICC	Intraclass correlation coefficient
κ	Cohen’s kappa coefficient
LBP	Lower back pain
MCs	Modic changes
ML	Machine learning
MRI	Magnetic resonance imaging
OR	Odds ratio
PROMs	Patient-reported outcome measures
r	Pearson correlation coefficient
R^2^	Coefficient of determination
ResNet	Residual Network
ResNet18	18-layer Residual Network
RR	Risk ratio
SNR	Signal-to-noise ratio
SPECT	Single-photon emission computed tomography
SPECT-CT	Single-photon emission computed tomography–computed tomography
SSD	Single-shot multibox detector
SymTC	Symmetric Transformer–CNN
U-Net	U-shaped convolutional neural network architecture
