# Supplementary material for: Artificial Intelligence in Spine Neuroimaging: Diagnostic and Prognostic Utility of Novel Biomarkers in Lower Back Pain
Source: J Clin Med. 2026 Jun 9;15(12):4447. doi: 10.3390/jcm15124447 (PMC13300810; doi:10.3390/jcm15124447)
Supplement: Supplementary file 1 [file jcm-15-04447-s001.zip › jcm-4257773-supplementary.pdf]

Supplementary Table S1. Structured quality/risk-of-bias appraisal, translational evidence level, and interpretive role of representative AI applications in spine imaging

For this narrative review, appraisal was qualitative and design-level. Each study was reviewed for validation design, data source, endpoint relevance, external validation, outcome linkage, and main bias/indirectness concerns. The appraisal is not a formal GRADE, QUADAS-2, PROBAST, ROBINS-I, or Cochrane risk-of-bias assessment.

**Study / reference**	**Task / modality**	**Design / validation**	**Key findings**	**Quality / risk-of-bias appraisal**	Evidence level / interpretive role
Bash et al. [27]	DL image enhancement/reconstruction; spine MRI	Prospective, randomized, multicenter, multireader technical validation against standard-of-care imaging	40% scan-time reduction maintained diagnostic integrity/image quality, with perceived SNR and artifact benefits.	Low risk for the technical endpoint; no patient-centered or management endpoint.	A (technical/measurement support)
Kashiwagi et al. [22]	DL reconstruction transferability; lumbar 1.5T MRI	Technical feasibility study applying a model trained on 3T brain/knee MRI to lumbar MRI	Supported approximately one-third scan-time reduction without clear loss of image quality.	Moderate risk/indirectness due to small technical design and domain transfer; no outcome validation.	C-D (technical feasibility)
Sun et al. [23]	High-resolution DL reconstruction; 3D lumbar MRI	Technical image-quality validation	3D DL-reconstructed T2-weighted FSE MRI improved perceived image quality with similar interobserver agreement for graded findings.	Moderate risk/indirectness; image-quality rather than clinical endpoint.	C (technical/measurement support)
Yeoh et al. [25]	DL noise reduction/edge sharpening; low-dose lumbar CT	Retrospective pilot technical validation in lumbar spine CT	DL reconstruction reduced noise while preserving/enhancing edge sharpness in low-dose CT images.	Moderate-to-high risk due to pilot design and technical endpoints; generalizability limited.	C-D (technical feasibility)
Greffier et al. [26]	DL reconstruction for dose optimization; lumbar CT	Phantom technical validation	Reported dose reduction up to 72% while maintaining detectability/image quality in the phantom setting.	High indirectness: phantom evidence does not establish patient-level performance.	D (phantom/technical)
Wang et al. [31]	Improved Attention U-Net segmentation; lumbar MRI	Retrospective technical segmentation validation	Improved lumbar spine segmentation performance versus comparator methods.	Moderate-to-high risk: segmentation endpoint, limited external validation, and no clinical outcome linkage.	C (technical/measurement support)
Hess et al. [33]	Multi-tissue segmentation/biomechanical modeling; lumbar MRI	Retrospective technical validation in BACPAC clinical MRI data	Automated multi-tissue segmentation supported quantitative feature extraction and subject-specific biomechanical modeling.	Moderate risk: clinically relevant workflow, but technical validation predominates.	C (technical/measurement support)
Niemeyer et al. [41]	CNN Pfirrmann grading; lumbar MRI	Large multicenter retrospective validation	1599 patients/7948 discs; reported kappa 0.92, sensitivity 90.2%, precision 92.5%, mean absolute error 0.08 grades.	Lower risk for classification reproducibility; retrospective labels and absence of outcome linkage remain limitations.	B (diagnostic classification)
Sayed et al. [42]	Cascade CNN disc localization/herniation detection; lumbar MRI	Retrospective algorithm validation	Sequential disc localization and classification yielded high reported disc-level classification performance.	Moderate-to-high risk: internal retrospective validation and limited prospective/external evidence.	C (diagnostic assistance)
Lu et al. [44]	DeepSPINE segmentation, disc-level designation, stenosis grading; lumbar MRI	Retrospective development/validation using 4075 patients and 22,796 disc levels	Automated stenosis classification reported average accuracies of 70.6% for central canal and 67.1% for foraminal stenosis.	Moderate risk: large dataset but retrospective, report/label dependent, and moderate ordinal performance.	C (diagnostic assistance)
Jamaludin et al. [51]; Ishimoto et al. [46]	SpineNet classification/evidence visualization; lumbar MRI	Retrospective model validation with epidemiologic application in the Wakayama Spine Study	Automated MRI grading showed useful dichotomous agreement, with weaker fine-grained ordinal performance.	Moderate risk: scalable method but dependent on reference labels and task simplification.	B-C (diagnostic assistance)
Hallinan et al. [54]	DL stenosis classification; lumbar MRI	Large retrospective validation against subspecialist radiologist readings	Comparable agreement with subspecialists for central canal and lateral recess stenosis; slightly lower agreement for foraminal stenosis.	Moderate risk: strong retrospective diagnostic-assistance evidence; prospective impact untested.	C (diagnostic assistance)
Gao et al. [50]	Voxel-wise Modic-change mapping; lumbar MRI	Retrospective technical validation in Modic-annotated lumbar MRI	Interpretable voxel-wise mapping preserved mixed-lesion tissue context.	Moderate-to-high risk: small/specialized dataset and limited routine-clinical validation.	C (technical/measurement support)
Liu et al. [53]	SSD + ResNet18 Modic-change detection/classification; lumbar MRI	Retrospective detection/classification validation	Reported approximately 86% accuracy and kappa approximately 0.7 for Modic detection/classification.	Moderate risk: annotation-dependent retrospective evidence.	C (diagnostic assistance)
Tomita et al. [55]	Deep neural network vertebral fracture detection; routine CT	Retrospective validation in 1432 CT scans, with held-out testing	Reported 89.2% accuracy and 90.8% F1 score; human readers had higher specificity.	Moderate risk: appropriate held-out testing, but retrospective and not implementation/outcome based.	C (diagnostic assistance)
Burns et al. [56]	Compression-fracture detection/classification and bone-density estimation; CT	Retrospective diagnostic validation	Reported sensitivity 95.7%, low false-positive rate, and weighted kappa 0.90 for Genant classification.	Moderate risk: strong technical/diagnostic performance; prospective workflow benefit not shown.	C (diagnostic assistance)
Murata et al. [57]	Vertebral fracture detection; thoracolumbar radiographs	Retrospective diagnostic validation in 300 patients	Reported accuracy 86% and sensitivity 84.7%, with comparison against orthopedic readers.	Moderate risk: balanced retrospective dataset; external/prospective validation limited.	C (diagnostic assistance)
Small et al. [59]	Cervical spine fracture detection; CT	Retrospective diagnostic validation in 665 CT examinations	Reported CNN accuracy 92%, sensitivity 76%, and specificity 97%; radiologists had higher sensitivity.	Moderate risk: useful triage evidence, but sensitivity below expert readers and no outcome endpoint.	C (diagnostic assistance/safety support)

Study / reference	Task / modality	Design / validation	Key findings	Quality / risk-of-bias appraisal	Evidence level / interpretive role
Giaccone et al. [15]	Automated paraspinal muscle morphology/composition; lumbar MRI	Retrospective/technical workflow validation in patients with LBP	U-Net-based segmentation quantified muscle area and fatty infiltration; reported Dice >95% and ICC 0.89 for area estimates.	Moderate risk: strong measurement reproducibility; causal and outcome evidence limited.	C (technical/measurement support)
D'hooge et al. [61]	Paraspinal muscle composition; recurrent unilateral LBP	Observational controlled MRI association study	Fatty infiltration was increased at lower lumbar levels despite no significant CSA differences.	Moderate risk: clinically relevant association, but observational and not an AI validation study.	B-C (contextual prognostic association)
Song et al. [65]	Whole sagittal spine segmentation/spinopelvic parameter analysis; radiographs	Retrospective technical validation	Automated X-ray segmentation enabled spinopelvic parameter evaluation.	Moderate risk: measurement-support evidence; limited nonspecific-LBP outcome validation.	C (technical/measurement support)
Galbusera et al. [66]	Automated radiological analysis of spinal disorders/deformity; radiographs	Retrospective technical validation	Automatically determined spine shape and calculated posture/anatomic parameters on biplanar radiographs.	Moderate risk/indirectness: deformity-oriented technical evidence, not LBP outcome validation.	C (technical/measurement support)
Vega-Alvear et al. [74]	Functional imaging for facet-intervention response; SPECT/SPECT-CT	Systematic review/meta-analysis; six studies, n = 308	Positive SPECT findings predicted pain relief after facet interventions (RR 2.06, 95% CI 1.54-2.75); SPECT-CT evidence was less consistent.	Moderate risk: outcome-linked but heterogeneous and not AI-derived.	A-B (contextual outcome-linked comparator; not AI-derived)
Halabian et al. [81]	ML in degenerative cervical myelopathy; contextual evidence	Conference abstract/critical review	Identified ML prognostication as an emerging area in DCM.	High risk/indirectness: abstract-level evidence outside LBP and not a full peer-reviewed validation study.	D (contextual; indirect)
Guan et al. [79]; Jia et al. [80]	Non-imaging risk factors for multimodal models	Mendelian-randomization meta-analysis and occupational-risk meta-analysis	Supported inclusion of lifestyle and occupational variables in multimodal risk frameworks.	Moderate indirectness: relevant risk context, but not imaging-AI validation.	B (contextual risk evidence)

Abbreviations: AI, artificial intelligence; CLAIM, Checklist for Artificial Intelligence in Medical Imaging; CNN, convolutional neural network; CSA, cross-sectional area; CT, computed tomography; DCM, degenerative cervical myelopathy; DL, deep learning; ICC, intraclass correlation coefficient; LBP, low back pain; ML, machine learning; MRI, magnetic resonance imaging; RR, risk ratio; SPECT, single-photon emission computed tomography; STARD-AI, Standards for Reporting Diagnostic Accuracy Studies-Artificial Intelligence.
